# Double Mesiodens in the Mixed Dentition of Non-syndromic North-Indian Patients: A Case Series

**DOI:** 10.7759/cureus.54161

**Published:** 2024-02-14

**Authors:** Supriya Bhatara, Mousumi Goswami, Shivangani, Bushra Rahman, Abhilash Gogoi

**Affiliations:** 1 Department of Paediatric and Preventive Dentistry, ITS Dental College, Hospital and Research Centre, Greater Noida, IND

**Keywords:** malocclusion, diagnosis [mesh], supernumerary [mesh], tooth abnormalities [mesh], mesiodens

## Abstract

The presence of double mesiodens or mesiodentes, i.e., two supernumerary teeth in the maxillary midline, presents unique challenges in mixed dentition. Common clinical manifestations include delayed eruption, midline diastema, and occlusal disturbances, leading to complications such as root resorption, pathological migration of tooth, crowding, cyst formation, and malocclusion. Mesiodens can be associated with several syndromes, like cleidocranial dysplasia, familial adenomatous polyposis, trichorhinophalangeal syndrome, type I, Rubinstein-Taybi syndrome, and Nance-Horan syndrome, among others. It can also be secondary to trauma, hyperactivity of the dental lamina, and a combination of genetic and environmental factors, but its etiology continues to be idiopathic.

Double mesiodens are relatively rare, so this clinical observation aimed to highlight five such cases of double mesiodens in mixed dentition in non-syndromic children and adolescents. Additionally, a literature search reporting cases of double mesiodens in the mixed dentition was done, and the results were tabulated. Clinicians should be able to identify indications of supernumerary teeth, specifically deviations in the eruption pattern. Appropriate investigations and timely intervention are essential to reducing complications that may arise in the developing dentition.

## Introduction

Supernumerary teeth are those in addition to the normal series of primary or permanent dentition. Hyperdontia resulting from one or more extra teeth can result in complications and deviations from normal dentition and development. The prevalence of supernumerary teeth [1,2] in the general population is 0.1%-3.8%. Supernumerary teeth can be of various types, depending on chronology, location (topography), morphology, and orientation. Mesiodens, supernumerary teeth occurring between the two central incisors, are the most common form of supernumerary teeth, accounting for 30% of the incidence [3-5]. Their appearance ranges from a simple conical shape to a crown structure with multiple tubercles. Mesiodens may occur as single, multiple, unilateral, bilateral, or rarely inverted. The presence of multiple mesiodens teeth is rare and is called 'mesiodentes’ [6].

The etiology of mesiodens remains unclear; however, various theories, such as the genetic basis, the dichotomy theory, the environmental factors, and the hyperactivity theory, which is the restricted, local, and independent increase in the activity of dental lamina, among many, have been described, out of which the hyperactivity theory seems to be the most acceptable etiological factor [4]. Some of the commonly associated syndromes with supernumerary teeth include cleidocranial dysplasia, familial adenomatous polyposis, trichorhinophalangeal syndrome, type I, Rubinstein-Taybi syndrome, Nance-Horan syndrome, Opitz BBB/G syndrome, Oculofaciocardiodental syndrome, and autosomal dominant Robinow syndrome [7].

A paucity of literature exists regarding double mesiodens or mesiodentes in non-syndromic patients. The presence of supernumerary teeth can lead to various pathological conditions. Commonly associated complications with supernumerary teeth include delayed or impaction of adjacent tooth/teeth, malposition or rotation of adjacent teeth, a diastema, and the formation of dentigerous cysts [4].

This article aims to report a case series of the rare occurrence of double mesiodens/mesiodentes in children and adolescents. Additionally, a literature search on electronic databases, i.e., PubMed, EBSCO, Google Scholar, SpringerLink, and manual searching of references with the relevant search terms (mesiodens, double mesiodens, supernumerary, dental anomalies, mixed dentition, children), was conducted with no restrictions on time or language.

## Case presentation

The following case series, prepared per the CAse REport (CARE) guidelines (Appendix 1), is a clinical observation of children presenting to the Department of Paediatric and Preventive Dentistry, ITS Dental College, Hospital and Research Centre, Greater Noida, between 2020 and 2023.

Case 1

**Figure 1:**
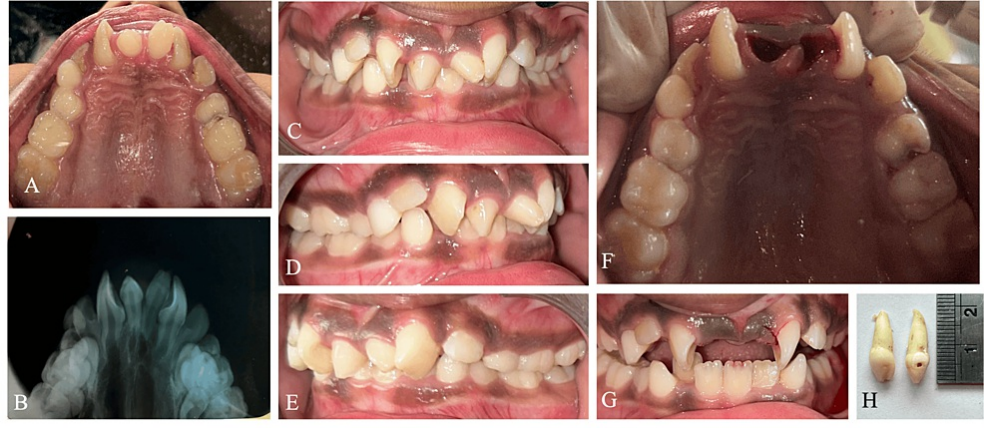
Case 1 A: pre-operative maxillary occlusal view; B: pre-operative maxillary occlusal intraoral periapical radiograph (IOPA); C: pre-operative frontal view; D: pre-operative right lateral view; E: pre-operative left lateral view; F: immediate post-operative maxillary occlusal view; G: post-operative frontal view; H: extracted mesiodentes

An eight-year-old Indian boy reported the chief concern of rotated teeth in the upper front tooth region for a year. The clinical examination revealed a non-syndromic child with a complete complement set of teeth in the mixed dentition. Two mesiodens were present in the midline, resulting in the rotation of 11 and 21 (Figure 1). The occlusion and arrangement of anterior teeth were disturbed secondary to the space deficiency created by the two central mesiodentes. The radiographic diagnosis included an orthopantomogram (OPG) and a maxillary occlusal intraoral radiograph. No other supernumerary teeth or abnormalities were observed in the OPG examination. The occlusal radiograph revealed two separate twin mesiodens, which were conical in shape. The root formation was incomplete in both teeth, and the periodontal ligament shadow around both teeth ruled out ankylosis. The treatment plan included the extraction of the mesiodentes, followed by orthodontic alignment of the teeth. As the child was apprehensive and had a lower pain threshold, the extraction was performed under infiltration and nasopalatine nerve block with 4% articaine (Septanest, Septodont, USA), and hemostasis was achieved with the help of pressure packing with cotton gauze. 

Case 2

**Figure 2:**
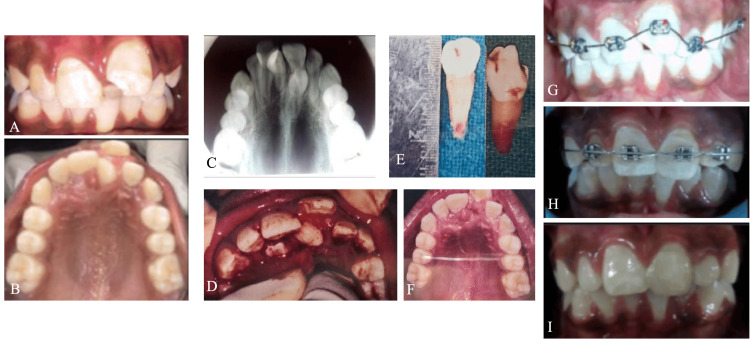
Case 2 A: pre-operative frontal view; B: pre-operative maxillary occlusal view; C: pre-operative maxillary occlusal intraoral periapical radiograph (IOPA); D: flap raised and extraction of mesiodentes; E: extracted mesiodentes; F: post-operative maxillary occlusal view; G,H: two-by-four appliance therapy; I: post-operative frontal view

An eleven-year-old girl reported maligned anterior teeth for three years and, upon a clinical examination, showed a palatally placed mesiodens with a buccal displacement of 21 with chalky white intrinsic stains due to dental fluorosis. An occlusal radiograph of the maxilla displayed an impacted mesiodens with respect to 11. Under a 2% lignocaine nasopalatine block with infiltration of 11 and 21, a mucoperiosteal flap was raised to expose the impacted supernumerary tooth, and both the mesiodentes were extracted, followed by self-resorbable sutures. Two-by-four appliance therapy (placing molar bands on 16 and 26), bonding brackets on maxillary anteriors, and sequentially changing arch wires from 0.012 to 0.018 were done for the alignment of 21 and 11, followed by finishing with composite restoration with respect to 21 (Figure 2).

Case 3

**Figure 3:**
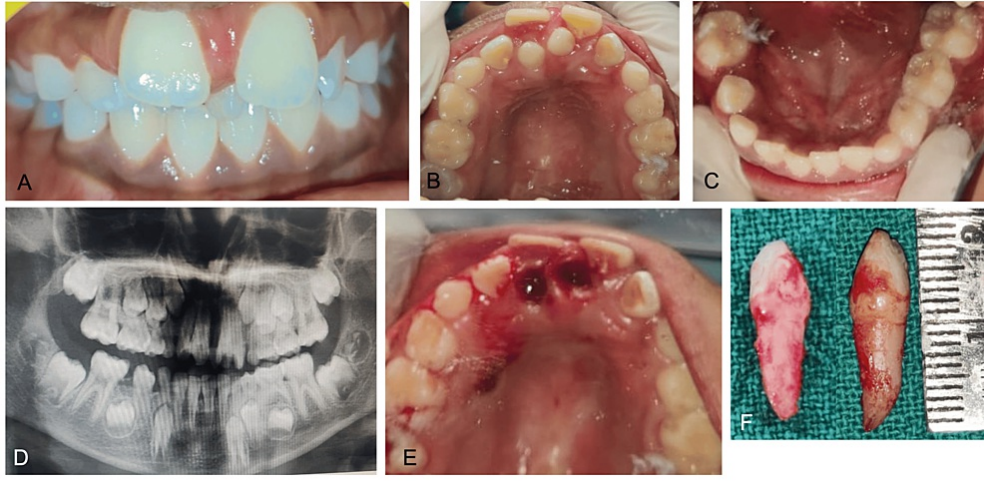
Case 3 A: pre-operative frontal view; B: pre-operative maxillary occlusal view; C: pre-operative mandibular occlusal view; D: pre-operative orthopantomogram (OPG); E: maxillary occlusal view; immediately post-extraction; F: extracted mesiodentes

An eight-year-old boy reported spacing between his upper front teeth for the last two years. The clinical examination revealed a midline diastema with a labially positioned 11 and a rotated 21 with an extra tooth between the two from the frontal view. Two mesiodentes present palatally were observed, which resulted in the labial displacement of both 11 and 21. Radiographic diagnosis via orthopantomography revealed two non-ankylosed mesiodens in the midline with no other associated pathologies. The treatment rendered was extractions under infiltration and nasopalatine nerve block with 2% lignocaine, followed by the initiation of two-by-four appliance therapy by placing molar bands on the first permanent molars and brackets bonded on 11, 21, 12, and 22; the arch wires, starting from gauge 0.012, were sequentially changed to 0.018 (Figure 3).

Case 4

**Figure 4:**
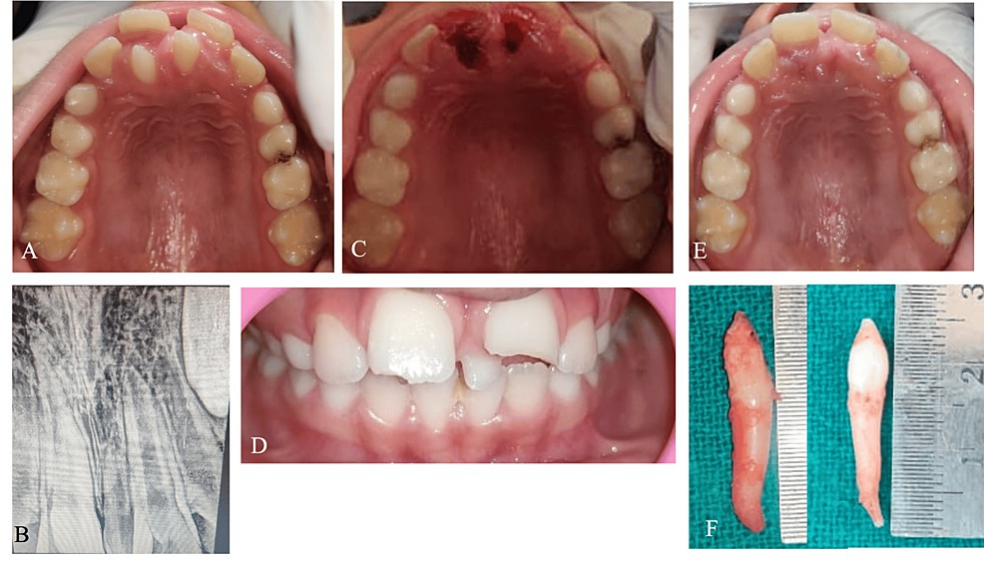
Case 4 A: pre-operative maxillary occlusal view; B: pre-operative intraoral periapical radiograph (IOPA); C: maxillary occlusal view, immediately post-extraction; D: pre-operative frontal view; E: maxillary occlusal view, post-healing; F: extracted mesiodentes

A nine-year-old boy reported a disturbance behind his upper front teeth for three years. The clinical examination revealed a midline diastema with a labially displaced and proclined 21 and an extra tooth between the two. Two mesiodentes present palatally with respect to 11 and 21 were observed. A radiographic diagnosis included an intraoral periapical radiograph (IOPA), which revealed two mesiodentes with no evidence of ankylosis (Figure 4). Extraction of the mesiodentes followed by two-by-four therapy was planned. The extractions were performed under 2% lignocaine infiltration and nasopalatine nerve block, and hemostasis was achieved with the help of a pressure pack. 

Case 5

**Figure 5:**
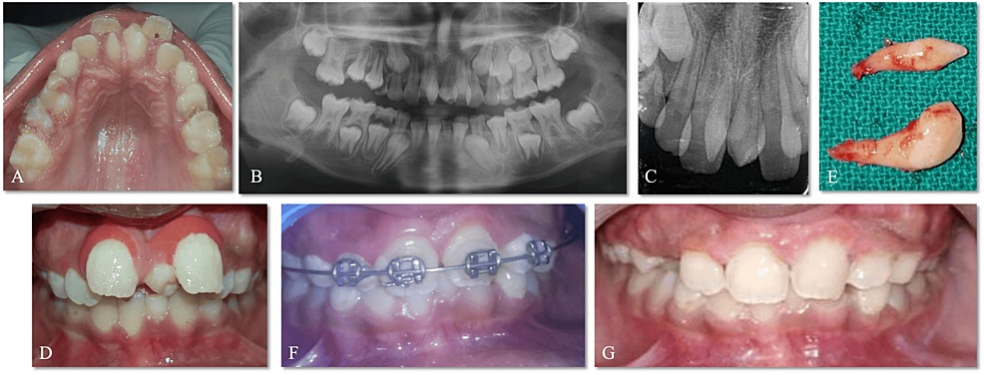
Case 5 A: pre-operative maxillary occlusal view; B: pre-operative orthopantomogram (OPG); C: pre-operative intraoral periapical radiograph (IOPA); D: pre-operative frontal view; E: extracted mesiodentes; F: two-by-four appliance therapy; G: post-operative frontal view

A nine-year-old girl reported the chief concern of protruding upper front teeth with spacing, which did not resolve by itself. The clinical examination revealed a midline diastema with labially displaced and proclined 21 and 11 with mesiodentes palatally. The OPG and an IOPA revealed two mesiodentes with no evidence of ankylosis (Figure 5). Extraction of the mesiodentes under 2% lignocaine infiltration and nasopalatine nerve block, followed by two-by-four therapy by placing molar bands on the maxillary first permanent molars and bonding brackets on the maxillary permanent incisors, which were followed by sequentially changing arch wires from 0.012 to 0.018, was done.

## Discussion

Although mesiodens are frequently encountered as an incidental discovery, the occurrence of double mesiodens remains an uncommon finding and can often evade detection due to a lack of awareness, resulting in the adoption of inappropriate imaging techniques and treatment approaches. It can, however, lead to various complications and should be addressed to ensure proper oral health, occlusion, and aesthetics. Treatment of double mesiodens typically involves either retention with observation or extraction of the supernumerary teeth. The timing of intervention may vary depending on the specific case, the root formation of the adjacent teeth, and the patient's age [8]. In all the cases discussed above, mesiodentes disturbed the occlusal harmony and were progressively discomforting to the patients, thus leading to the treatment plan for immediate extraction. Therefore, it is often observed that patients display symptomatic attendance, so early intervention is not always possible, increasing the likelihood of complications. The disturbed occlusal characteristics of maxillary anterior teeth in children presenting during their early mixed dentition stages can be treated with two-by-four appliance therapy, which comprises bonds on the maxillary incisors, bands on the first permanent maxillary molars, and a continuous archwire change until desirable results are achieved. This can eliminate the malocclusion or limit its extent, therefore reducing the need for prolonged orthodontic treatment. This has been depicted in Cases 2 and 5. These frequent diagnoses of double mesiodens in this population may either be the result of the ethnic makeup of the population or the improved diagnostic methods.

**Figure 6:**
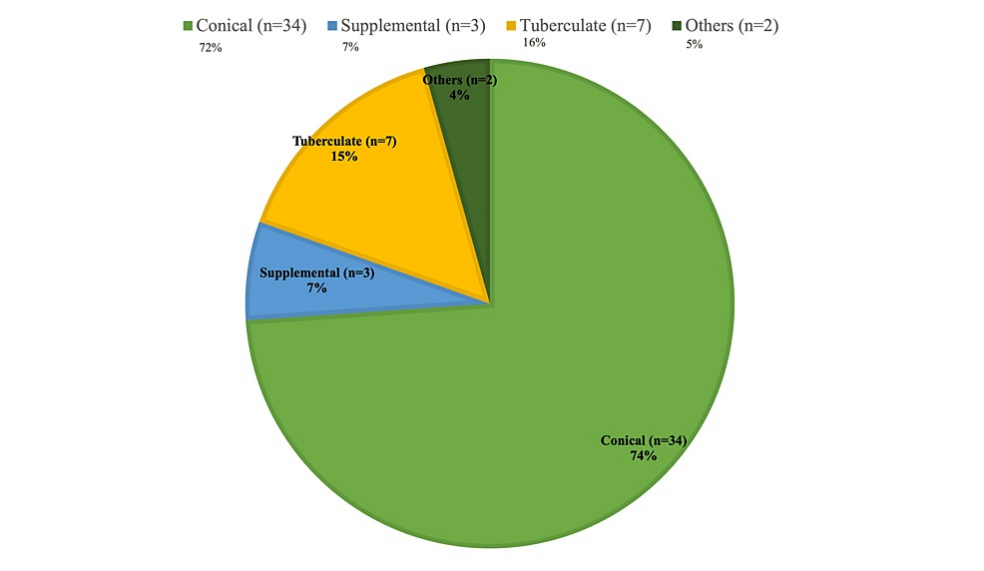
Morphology of mesiodentes reported in the studies Cases reported in the studies identified through the literature search [5,6,8,12-20]

**Figure 7:**
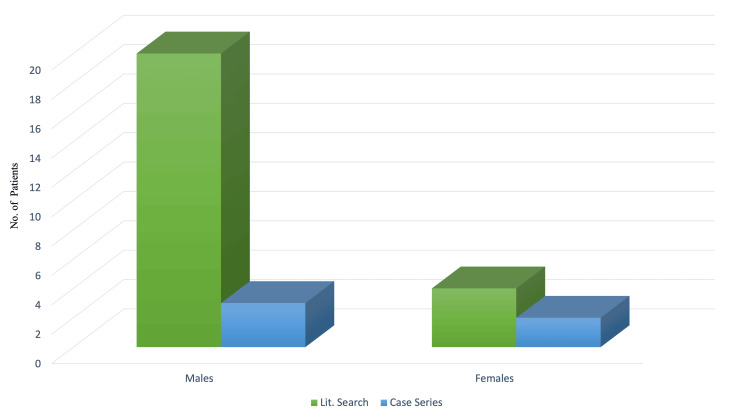
Comparison of genders for the occurrence of mesiodentes Gender distribution of mediodentes occurrence in the present report and the studies identified in the literature [5,6,8,12-20]

Out of the various heterogeneous morphological forms, the conical type is cited as the most prevalent type [9]. In our clinical observation and the literature search (Table 1), the most common morphological combination was a combination of the conical and tuberculate types in a single patient with mesiodentes. The conical type was, however, the most prevalent type of mesiodens, followed by the tuberculate type (Table 2). This was consistent with the findings of the literature search conducted, which found the conical type was the most prevalent type, followed by the tuberculate type (Figure 6). The literature reports that mesiodens is more prevalent in men when compared to women, with a ratio of 2:1 when it comes to non-syndromic occurrences [10,11], and a similar finding was observed in our clinical observation, where 3 out of 5 cases were males (Figure 7).

**Table 1:** Literature studies

Author (Year)	Number of reported cases	Patient		Eruption status	Position	Shape	Associated abnormalities	Aesthetic/Additional treatment
		Sex	Age (Years)					
Krishnappa S et al. (2014) [12]	One	F	16	Impacted	Inverted	Conical	Anterior crossbite	-
Sujlana A et al. (2017) [13]	Two	M	12	Erupted/Impacted	Straight/ Inverted	Conical	Trauma to anterior region at five years of age	Transnasal approach
		M	8	Erupted	Straight	Conical	Trauma to anterior region at four years of age, impacted 21 and 11	Spontaneous eruption of 21 and 11 at four Months
Rana SS et al. (2018) [14]	Three	M	10	Erupted	Straight	Conical	Malaligned upper anterior teeth	-
		M	9	Erupted/Impacted	Straight	Supplemental/Conical	Midline diastema	
		M	7	Erupted/Impacted	Straight	Tuberculate/Conical	-	-
Dinkar AD et al. (2007) [15]	One	F	14 y	Impacted	Inverted	Conical	Dentigerous cyst	Root canal treatment wrt 21 and 11
Canoglu E et al. (2009) [16]	One	M	8	Impacted	Inverted	Conical	Impaired occlusion	Self-correction of axial rotation of 11 and 21
Venkataraghavan K et al. (2011) [17]	One	M	13	Erupted	Straight	Conical	Impaired occlusion and speech dysfunction	Speech therapy
Gharote HP et al. (2011) [18]	Six	M	24	Erupted	Straight	Tuberculate	Impaired occlusion	Ellis class III and II fractures
		M	10	Impacted	Straight/Inverted	Conical/Tuberculate	Intraoral swelling	Radicular cyst wrt 21 and 22
		M	12	Erupted, fused with central incisor	Straight	Conical/ tuberculate/ fused with central incisor	Impaired occlusion	Not a true case of double mesiodens but three supernumerary teeth in the midline
		M	26	Erupted	Straight	Conical/Supplemental	-	-
		M	20	Erupted/Impacted	Straight/Inverted	Conical	Irritation to tongue	
		M	20	Partially erupted/Impacted	Straight/Inverted	Conical	Accidental finding (R/G)	Ellis class II fracture wrt 21 and 11, RCT wrt 11 and 21
Jafri SA et al. (2011) [19]	One	M	9	Partially erupted/Impacted	Straight/Inverted	Incisiform/globular with incomplete root formation	Impacted 21, unaesthetic appearance	Spontaneous eruption of 21 in two months
Sulabha AN et al. (2012) [20]	One	M	13	Partially erupted	Straight	Tuberculate	Mesiodentes with dens invaginatus	-
Gurgel CV et al. (2013) [5]	Two (Twins)	M	9	Impacted	Straight	Conical	Unerupted 21,11, over-retained 51	Hyrax palatal expander, eruption of 21,11 in 10 Months
		M	9	Impacted	Straight	Conical	Unerupted 21,11, over-retained 51 61	Hyrax palatal expander, eruption of 21, 11 in 12 Months
Omami M et al. (2015) [6]	One	F	8	Unerupted	Inverted/Straight	Conical/Supplemental	Delayed eruption of 11	Spontaneous eruption
Asha ML et al. (2015) [8]	One	M	10	Erupted	Straight	Conical	6 mm midline diastema	Orthodontic treatment
Koyama Y et al. (2023) [21]	Three (2 of double mesiodens)	M	9	Unerupted	Inverted	Conical		Altered eruption of lateral incisors, crowding
		M	7	Erupted/unerupted	Straight/Inverted	Conical/Not mentioned		

**Table 2:** Morphology of mesiodentes in cases 1 to 5 in the present report

Case	Sex	Age (years)	Shape of mesiodens (11)	Shape of mesiodens (21)
1	Male	8 Years	Conical	Conical
2	Female	11 Years	Tuberculate	Supplemental
3	Male	8 Years	Tuberculate	Conical
4	Male	9 Years	Conical	Conical
5	Female	9 Years	Conical	Tuberculate

Mesiodentes interfere with the eruption of permanent central incisors and may result in their impaction. Also, mesiodentes may erupt into the oral cavity or remain impacted [9]. Delayed eruption of associated teeth has been reported to occur in 28−60% of Caucasians with supernumerary teeth [11]. In our clinical observation, all the central incisors and the mesiodentes pairs had erupted into the oral cavity, and the time of reporting by the parents was symptomatic and only when they noticed significant orthodontic or aesthetic changes. 

If left untreated, mesiodens have been associated with dental irregularities, delayed emergence of permanent teeth, prolonged retention of deciduous teeth, and the potential formation of cysts [21]. Sometimes, undetected cases can go on to cause complications such as recurrent rhinitis due to the migration of mesiodens towards the nasal floor. A recent case report depicted bilateral inverted mesiodentes that had migrated to the floor of the nasal cavity in a 21-year-old [22]. A case report of the surgical extraction of mesiodens impacted in the lower nasal floor in a 20-year-old highlights the role of untreated mesiodens in the etiology of an intranasal tooth. An intranasal retained tooth can cause symptoms like facial pain, external nasal deformities, foul-smelling rhinorrhoea, recurrent epistaxis, nasal obstruction, and oronasal fistulae [23]. Interestingly, a case report of two inverted mesiodens resulting in an oronasal fistula in a 62-year-old man due to the development of infection around them highlighted that with age, the untreated mesiodens tend to migrate pathologically, i.e., inverted mesiodens tend to move closer to the nasal side with the aging of the patient while developing mesiodens tend to move farther away from the nasal side [24]. The literature search also reflected the occurrence of dentigerous cysts in untreated residents. These complications can complicate the treatment plan and increase the burden of the condition. These findings underscore the need for timely detection, correct diagnosis, and informed treatment plans. 

There have been a few studies citing the occurrence of mesiodentes, and their findings are highlighted (Table 1). Still, to the best of our knowledge, this is the first one highlighting five cases of double mesiodens, especially in mixed dentition in the north Indian population, thus providing evidence for further understanding this rare phenomenon.

## Conclusions

Insufficient knowledge and management skills can present a diagnostic challenge when dealing with double mesiodens or mesiodentes. The clinical observation showed a more prevalent occurrence in males and of the conical and tuberculate morphological types. The timing of the intervention should depend on a holistic care plan, taking into account the root development of the adjacent teeth and the associated complications. Pediatric dentists frequently serve as the initial healthcare providers for children and adolescents when it comes to addressing oral health concerns. Among these concerns, malocclusion and aesthetic considerations have gained increasing prominence. Therefore, it is imperative to acquire a comprehensive understanding of these relatively uncommon phenomena and possess substantiating evidence for treatment approaches. The objective of this clinical observation is to contribute to this knowledge by shedding light on the infrequent incidence of double mesiodentes.

## Appendices

Appendix 1 

**Table 3:** CAse REport (CARE) checklist

		Reporting item	Page number
Title			
	#1	The area of focus and “case report” should appear in the title	1
Keywords			
	#2	Two to five keywords that identify topics in this case report	1
Abstract			
Introduction	#3a	What is unique and why is it important?	1
	#3b	The patient’s main concerns and important clinical findings	
	#3c	The main diagnoses, interventions, and outcomes	
Conclusion	#3d	What are one or more “take-away” lessons?	1
Introduction			
	#4	Briefly summarize why this case is unique with medical literature references	1-2
Patient information			
	#5a	De-identified demographic and other patient information	Yes
	#5b	Main concerns and symptoms of the patient	Yes
	#5c	Medical, family, and psychosocial history including genetic information	Yes
	#5d	Relevant past interventions and their outcomes	Yes
Clinical findings			
	#6	Relevant physical examination (PE) and other clinical findings	Yes
Timeline			
	#7	Relevant data from this episode of care organized as a timeline (figure or table)	-
Diagnostic assessment			
	#8a	Diagnostic methods (PE, laboratory testing, imaging, surveys)	Yes
	#8b	Diagnostic challenges	Yes
	#8c	Diagnostic reasoning including differential diagnosis	-
	#8d	Prognostic characteristics when applicable	Yes
Therapeutic Intervention			
	#9a	Types of intervention (pharmacologic, surgical, preventive)	Yes
	#9b	Administration of intervention (dosage, strength, duration)	Yes
	#9c	Changes in the interventions with explanations	Yes
Follow-up and outcomes			
	#10a	Clinician and patient-assessed outcomes when appropriate	Yes
	#10b	Important follow-up diagnostic and other test results	-
	#10c	Intervention adherence and tolerability (how was this assessed?)	-
	#10d	Adverse and unanticipated events	-
Discussion			
	#11a	Strengths and limitations in your approach to this case	Yes
	#11b	Discussion of the relevant medical literature	Yes
	#11c	The rationale for your conclusions	Yes
	#11d	The primary “take-away” lessons from this case report	Yes
Patient perspective			
	#12	The patient can share their perspective on their case	-
Informed consent			
	#13	The patient should give informed consent	Yes
